# Some Thoughts on the Combination of Amalgam and Gold

**Published:** 1896-01

**Authors:** F. W. Knowlton


### Some Thoughts on the Combination of Amalgam and Gold.

BY DR. F. W. KNOWLTON.

Read before the Ohio State Dental Society, December 4th, 1895.

Amalgam and gold in combination as a filling material is indeed an old subject, and we wonder what more can be said regarding it; but the fact that it continues to be discussed seems to indicate that it has inherent qualities which are worthy of our consideration. Those who are conversant with the practical use of this material in their daily work need not be reminded of the many benefits to be derived by both patient and operator, and the thoughts expressed will be merely a repetition of that with which they are familiar, for I have not the hope of presenting any new idea or mode of procedure, but I am glad to give others the credit which is due them for what has been accomplished in this line.

Perhaps there are some who have not experienced for themselves its merits, whether from lack of faith in the results, or otherwise. If there be any such present, possibly there may be something said in this paper, or the discussion of it, that will induce them to give such a filling a fair trial, that they may know if there be any virtue in it. I hardly need state that the same care and judgment are necessary in the application of this as of all filling materials if we would attain the results which are possible. It is when we make a use of filling materials other than that for which they were intended that they are unable to withstand the criticisms passed upon them, and are condemned in consequence of a lack of proper discrimination. Many times this combination can be used and better results follow than when gold or amalgam alone are relied upon, and in not a few cases in which it would be impossible to obtain the desired end with any other material. The plea that two sittings are necessary in order to complete such a filling no longer holds, for with good alloy and Steurer's Plastic Gold such objections are overcome with entire success, although in some cases there is an advantage in allowing the amalgam to harden and complete with the gold at a subsequent sitting. The satisfaction of knowing that there is a perfect union between the amalgam and gold, giving you practically one material, is certainly something in its favor. I do not think that this kind of work will make slovenly operators of us, for the care necessary for its successful insertion is best realized by those who are familiar with its workings.

As an illustration, we will take a lower molar tooth, medium in texture, the proximal surface badly undermined, usually extending on the occlusal surface.

Under existing circumstances we may dislike to put in amalgam, and hesitate to insert a gold filling, but if we can combine the two, retaining the better properties of each and avoiding the less desirable ones, is it not a good practice to follow? In such a case the different parts of the cavity are prepared consonant to the metal it is to receive, the lower third, or possibly half of the cavity, depending somewhat on the size of the same, made suitable for an amalgam filling, the remainder of the cavity prepared for gold, this to be retained in place independent of the amalgam portion. Adjust the matrix securely, and at the lower third or half, as the case may be, perfectly, so there will not be the least danger of any of the amalgam being forced out around the matrix. Insert the amalgam in the usual way, filling the cavity rather full and then remove it down to the proper amount. In this way the portion that remains will be quite hard and free from excess of mercury. Insert the plastic gold immediately, forcing it on to the amalgam with large points at the start, continue adding to the gold until it finally overcomes the mercury, then complete the filling in the ordinary manner, using the same or any other gold you may prefer. Remove the matrix and finish as you would any gold filling, noting the ease with which you are able to dress down the filling at the cervical margin without any great annoyance to the patient. The galvanic action which takes place, as a result of the contact of the different metals, causes the oxides and sulphides to be deposited on the amalgam more abundantly than when it is used alone, consequently this portion of the filling becomes quite black, which to many is a commendable feature, preventing recurring delay at that vulnerable spot, the cervical margin, whether mechanically, by filling the dentinal tubules, chemically, therapeutically, or by all combined we leave to each one's choice, but this we do believe, that the results obtained justify the means. There is no bulging, crevicing, or disintegrating of the amalgam under the galvanic action, for it remains just as left after it receives the finishing touches. The gold portion of the filling serves us in its own good way, preserving its own and the margins of the tooth as only gold can, under the strains to which the teeth are subjected in this location during mastication; and that important part of the operation, that of reproducing the original contour of the tooth, which tends to preserve the health of the surrounding tissues and makes a presentable piece of work in the eyes of the patient, can best be accomplished with gold. There is a pronounced diminution in the number of troublesome cases caused by the extremes of heat and cold, more so than when either metal alone is used under like conditions, but this must not be taken as an argument that we need not use precaution. No tissues are apt to perform their normal function, unless free from irritation, and whatever aids us in maintaining such tranquility of nerve possesses a commendable feature. This is only one of the many valuable combinations which we are permitted to use in operative dentistry, and it does seem as though there are good grounds for the belief expressed by many of those prominent in our profession that this sort of filling will prove satisfactory to patient and operator, but only when coupled with that essential element of a dentist's make-up —judgment.

